# Overground gait adaptability in older adults with type 2 diabetes in response to virtual targets and physical obstacles

**DOI:** 10.1371/journal.pone.0276999

**Published:** 2023-09-13

**Authors:** Suzanne Martin, Simon B. Taylor, Blynn L. Shideler, Rajna Ogrin, Rezaul Begg

**Affiliations:** 1 Institute of Health Exercise and Sport, Victoria University, Melbourne, Australia; 2 School of Medicine, Stanford University, Stanford, California, United States of America; 3 Bolton Clarke Research Institute, Melbourne, Australia; Kennedy Krieger Institute/Johns Hopkins University School of Medicine, UNITED STATES

## Abstract

**Background:**

To step over an unexpected obstacle, individuals adapt gait; they adjust step length in the anterior-posterior direction prior to the obstacle and minimum toe clearance height in the vertical direction during obstacle avoidance. Inability to adapt gait may lead to falls in older adults with diabetes as the results of the effects of diabetes on the sensory-motor control system. Therefore, this study aimed to investigate gait adaptability in older adults with diabetes.

**Research question:**

Would diabetes impair gait adaptability and increase sagittal foot adjustment errors?

**Methods:**

Three cohorts of 16 people were recruited: young adults (Group I), healthy older adults (Group II), and older adults with diabetes (Group III). Participants walked in baseline at their comfortable speeds. They then walked and responded to what was presented in gait adaptability tests, which included 40 trials with four random conditions: step shortening, step lengthening, obstacle avoiding, and walking through. Virtual step length targets were 40% of the baseline step length longer or shorter than the mean baseline step length; the actual obstacle was a 5-cm height across the walkway. A Vicon three-dimensional motion capture system and four A.M.T.I force plates were used to quantify spatiotemporal parameters of a gait cycle and sagittal foot adjustment errors (differences between desired and actual responses). Analyses of variance (ANOVA) repeated measured tests were used to investigate group and condition effects on dependent gait parameters at a significance level of 0.05.

**Results:**

Statistical analyses of Group I (n = 16), Group II (n = 14) and Group III (n = 13) revealed that gait parameters did not differ between groups in baseline. However, they were significantly different in adaptability tests. Group III significantly increased their stance and double support times in adaptability tests, but these adaptations did not reduce their sagittal foot adjustment errors. They had the greatest step length errors and lowest toe-obstacle clearance, which could cause them to touch the obstacle more.

**Significance:**

The presented gait adaptability tests may serve as entry tests for falls prevention programs.

## Introduction

In older populations (aged 65 or older) there is an increased risk of falls in individuals with diabetes [1]. Diabetes is a metabolic condition in which defective insulin secretion, insulin resistance or both cause hyperglycaemia.

One of the complications in older people with diabetes is an increased risk of falls that has several consequences. The annual incidence of falls has been reported to be 39% [2]. Meanwhile, over 50% of older people with both types of diabetes have reported at least one injurious fall or two non-injurious falls a year [3]. Around 30–50% of these falls caused minor injuries, and 5–10% caused major injuries including fractured neck of femur [4]. Apart from fall-related injuries, falls greatly increase the cost of national health care. The reported direct annual medical cost was US$23.3 billion in the United States and US$1.6 billion in the United Kingdom [5]. Fall-related events account for 40% of nursing home placements and contribute to further increases in healthcare costs [6].

**Fig 1:**
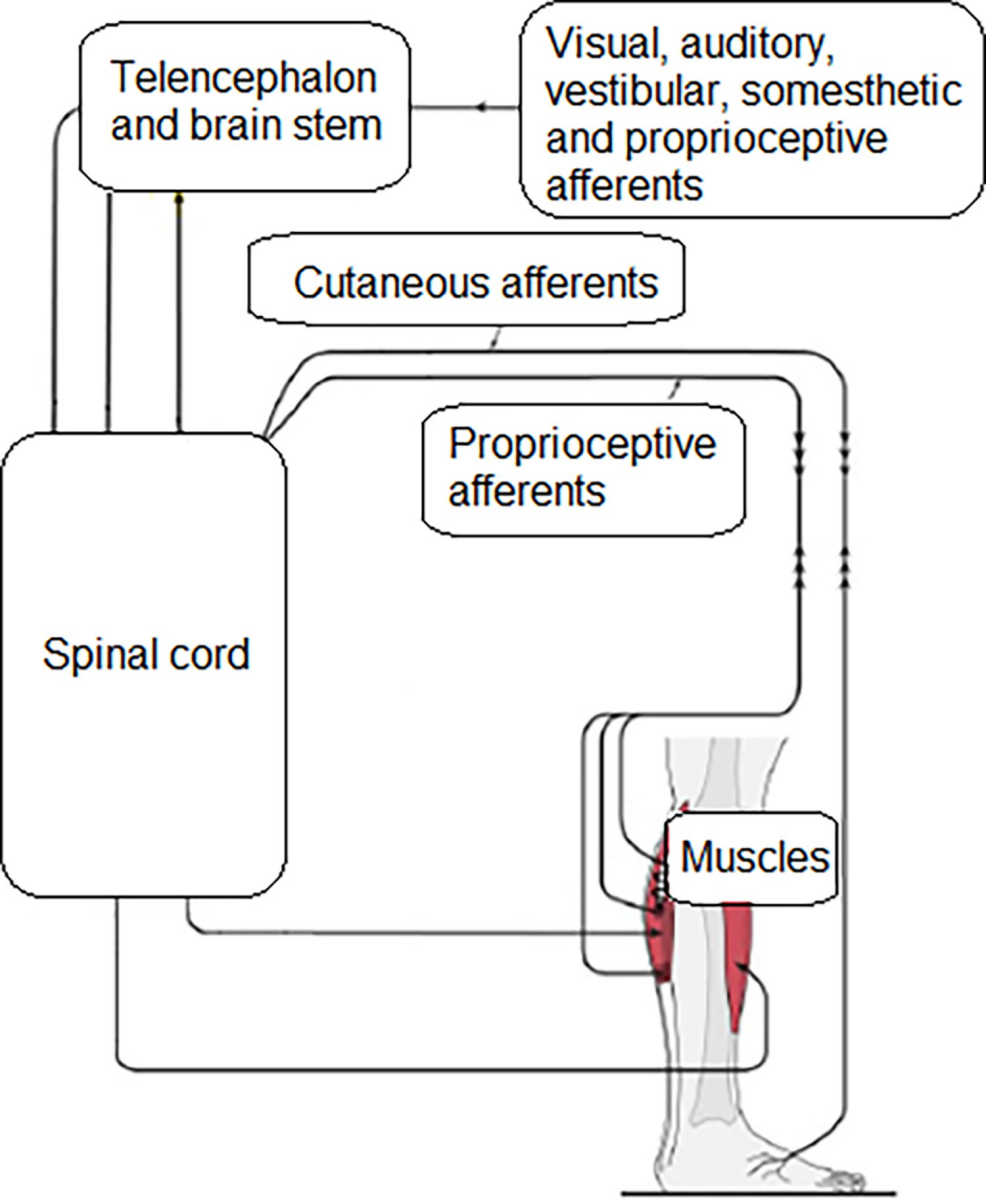
The central nervous system receives sensory information (afferent feedback) from several locations to update efferent copies of motor commands.

**Fig 2:**
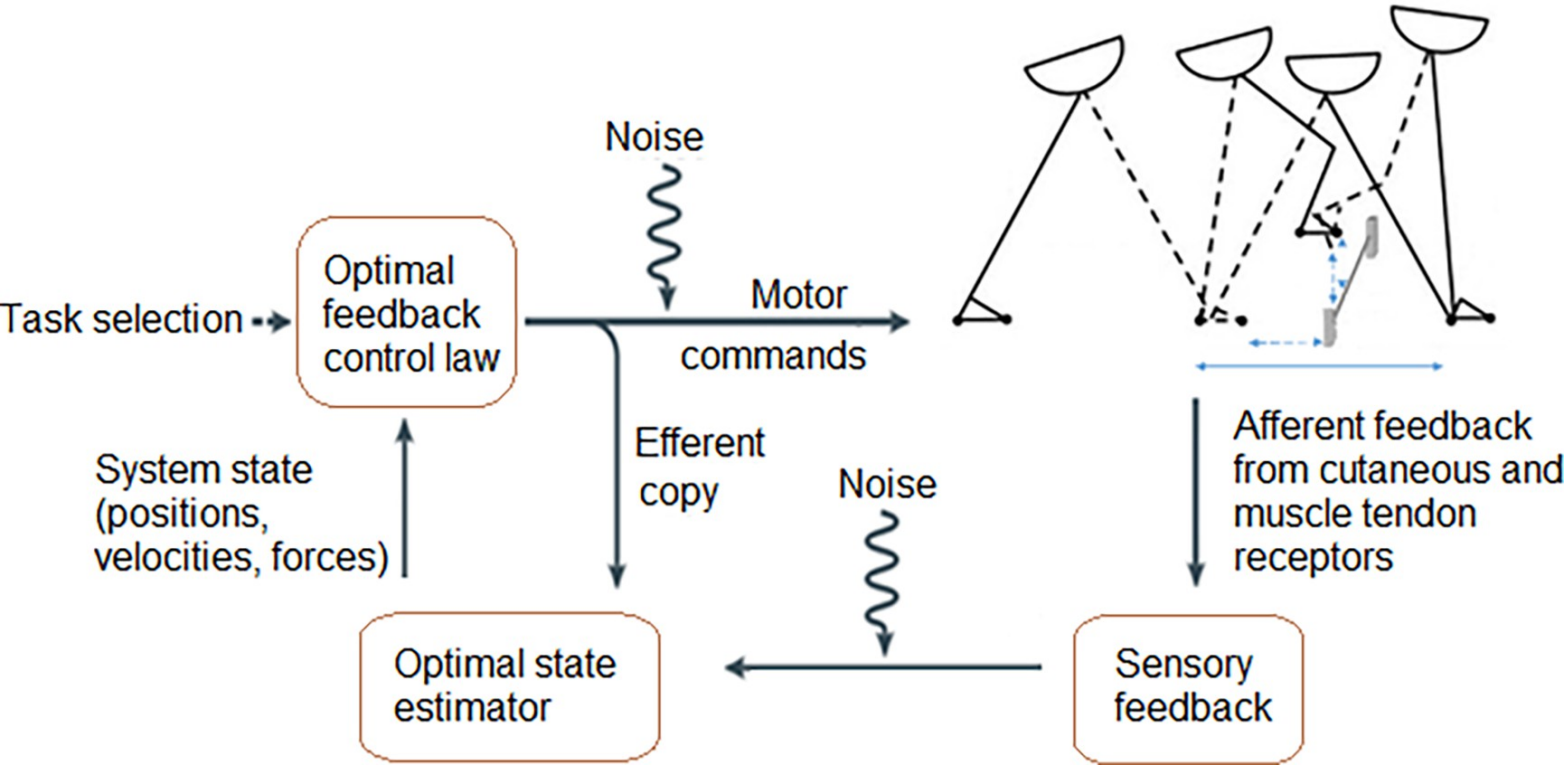
Optimisation of motor commands by the sensory-motor system. The sensory-motor control system produces and modulates locomotion using afferent feedback to adapt efferent copies of motor commands to external requirements for safe navigation.

Diabetes like some other pathological conditions may impair gait adaptability that is one of the reasons for falls in some neuromusculoskeletal conditions (stoke, Parkinson’s disease, lower limb amputation) [7–11]. Gait adaptability–adaptation of gait spatial temporal parameters in response to visual and auditory stimuli–occurs in response to external hazards such as obstacles. All elements of the sensory-motor control system (Fig 1) must work together to update efferent commands when a person avoids an obstacle. Any change in the sensory-motor control can reduce the accuracy of the response, and lead to a contact between the foot and the obstacle (Fig 2).

Longstanding hyperglycaemia changes the structure of distal nerves. The glycation of nerve tissues compromises the vascular supply by forming non-degradable advanced glycation products on the extracellular connective tissue matrix of nerves [12]. This reduces the blood flow and nerve conduction velocity [13], degenerates distal nerve fibres, and prevents nerve regeneration [14]. Previous investigators reported a decrease in walking speed and step length during normal walking, and a reduced toe-obstacle clearance during obstacle crossing in people with diabetes [15, 16]. In line with previous studies on people with other chronic conditions [8, 12, 21, 22], participants with diabetes did not touch any obstacles because they could see them a few steps a head.

Despite the prevalence of falls in older adults with diabetes, only a few studies [15–23] have investigated the effects of diabetes in response to obstacle avoidance. Although these studies reported that diabetes reduced toe-obstacle clearances, none of their participants touched obstacles [15, 16] because obstacles were always visible and participants could see obstacles from the starting point, so they could adapt the lengths of steps a few steps ahead to place obstacles in the middle of their crossing steps and avoid them. However, in dynamic environments, individuals must adapt gait on-demand to prevent falls and adverse outcomes. Such gait adaptation has yet to be studied in diabetic populations, which may partially contribute to the clinical population’s increased risk of falls. Accordingly, the present study aimed to compare biomechanical changes in gait adaptability tests among young adults, older adults without any health conditions (healthy older adults), and older adults with type 2 diabetes (T2D). It was hypothesised that T2D, ageing, and the combination of T2D and ageing would impair gait with and without challenges.

## Methods

### Participants

A priori power calculation estimated 48 participants in three group would be required to detect an effect size of 0.66 with 95% power. Therefore, 16 young adults, 16 healthy older adults, and 16 older adults who had lived with T2D for at least five years without having diabetic complications (i.e., foot ulcer) were recruited. All participants had no cognitive function impairment (The mini-mental state examination (MMSE) [24] score ≥ 23), vision impairment a history of falls in a year before their participation, or ageing- and diabetes-related neuropathy (The Michigan neuropathy screening instrument [25] score ≤ 3).

All procedures were approved by the Victoria University Human Research Ethics Committee (HRE17‐194). All participants provided written informed consent and visited the Institute for Health and Sport, Victoria University, Melbourne, Australia to participate in the research study.

### Experimental set-up and procedure

A three-dimensional motion analysis system (VICON, Oxford, UK) with 14 cameras and four A.M.T.I forces plates (Advanced Mechanical Technology Inc., Watertown, Mass., USA) was used to record gait biomechanics. The motion analysis system tracked four total reflective markers attached bilaterally to the distal part of the first toe and the distal posterior aspect of bisection of the heel of each shoe. The system recorded kinetic and kinematic gait data when participants walked for eight metres at their self-selected speed in baseline without any challenges and in adaptability tests with four conditions.

**Fig 3:**
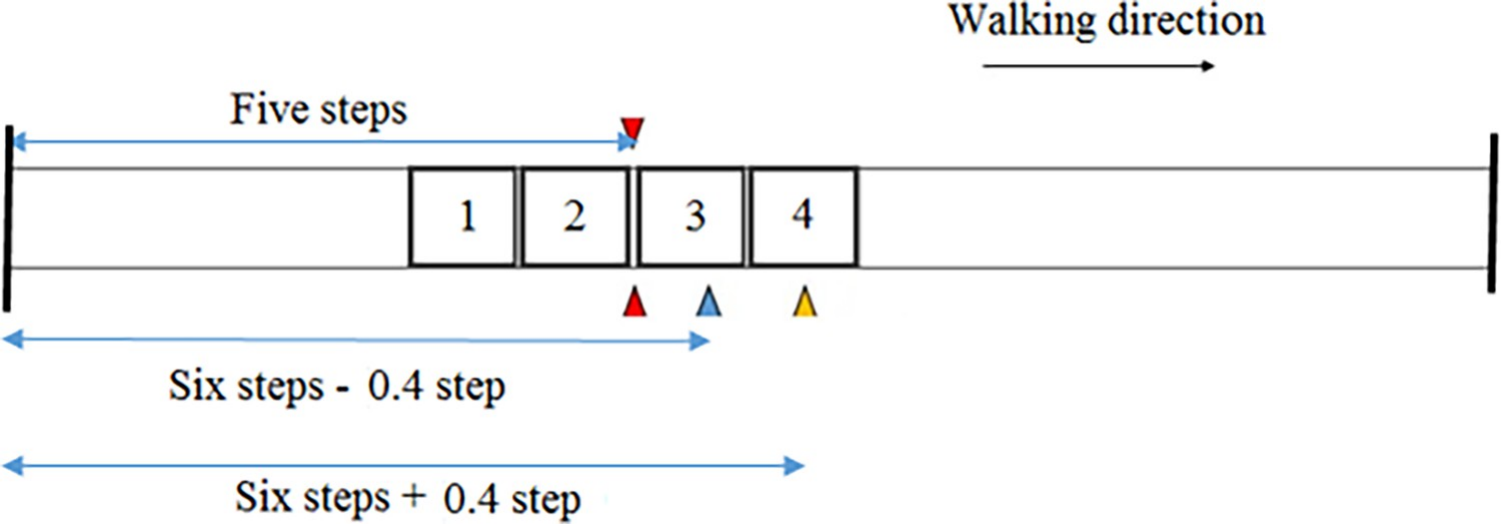
Overhead view of the experimental setup in the foot displacement adaptation test in the walkthrough condition. Red triangles present the locations of two servomotors which were triggered at the same time. The blue and yellow triangles present the locations of two laser beam projectors for short and long step targets (SSL and LSL targets). Two black lines present the starting and finishing points.

Participants walked in a baseline walking condition (6 trials), and then completed adaptability tests (40 trials). In adaptability tests, participants walked at their self-selected speed and responded to one of four challenging conditions: shortening the target step, lengthening the target step, crossing an obstacle, or walking through without any change. The difference between walking through and baseline walking was the knowledge that a task (step length adjustment or obstacle crossing) might be triggered. Participants had to prioritise the task in adaptability tests. Participants were blinded to the gait adaptability challenge during in adaptability tests; challenges were randomly assigned and presented to participants two steps ahead during overground walking (Fig 3).

**Fig 4:**
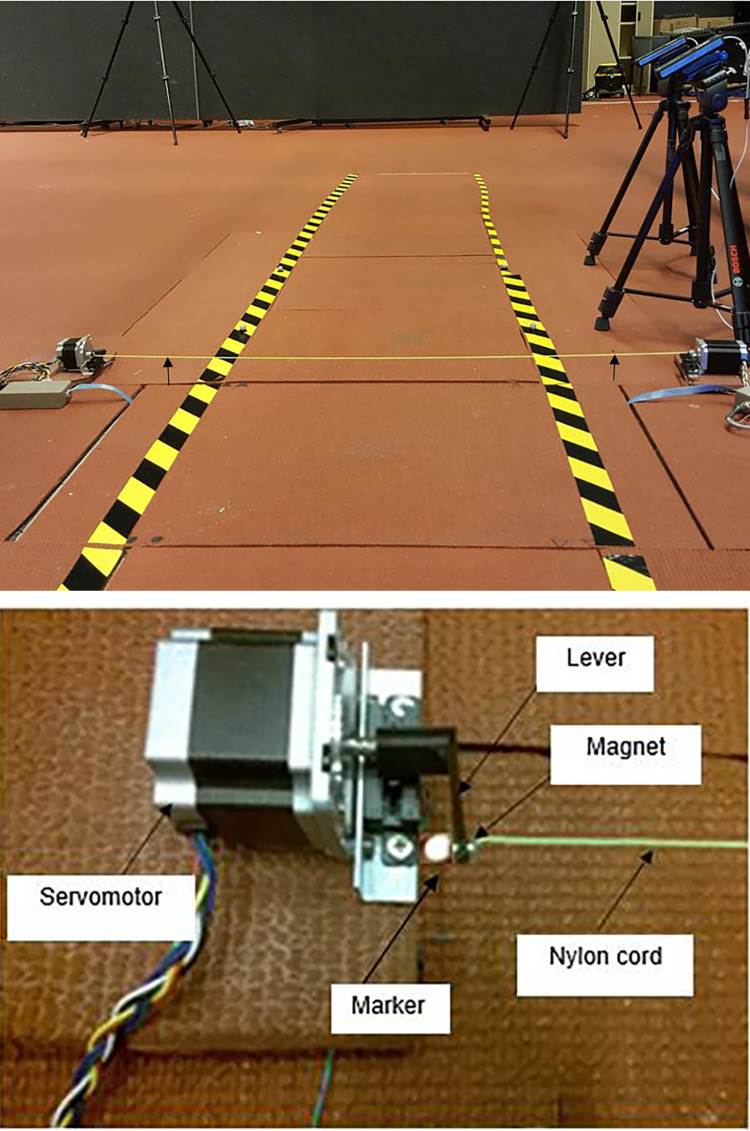
The 5-cm low height obstacle was presented sometimes in gait adaptability tests. The yellow nylon cord was held at a 5-cm height when the synchronised servomotors were turned on by touching the first force platform (A). A nylon cord was attached on each end to a servomotor’s lever by a small magnet and sat on the ground when participants stood behind the starting points (five steps away) and was randomly lifted in the air to show a 5-cm height when participants were two steps away (B).

Short and long step targets (SSL and LSL targets) were projected on the walkway by using laser beams virtually, whereas the obstacle was a fine cord that was lifted for 5 cm (low height) by two servomotors (Fig 4). A slight touch of a foot during crossing the obstacle immediately caused the cord attached to the servomotors to become detached. Thus, the forward swing of the leg did not affect balance if participants could not avoid the obstacle.

Step targets were subject-specific. In baseline, the mean step length of an individual was calculated. Step targets were scaled to a participant’s baseline step length (SL) and were 0.6 × baseline SL and 1.4 × baseline SL [26]. The locations of stepping targets and the obstacle were adjusted for each subject based on his/her mean step length in baseline (Fig 3).

Participants were not instructed on how to respond in the experimental adaptability tests. However, they were instructed how to respond to the presentation of each condition in a practice session. In adaptability tests participants walked for four steps, and in the fifth step when the foot landed on the first platform (Fig 3), one of the conditions was presented and participants had to respond to the condition in the target step.

For each participant, the locations of blue and yellow lines were adjusted at the end of the baseline walking trial. Two laser projectors were switched off when participants stood at the starting point. They were unaware of the location of the projection of a line during the experimental trials. Only examiners knew when and which laser projector would be triggered. Participants walked and responded as they saw a task two steps ahead.

Experimental trials were deemed successful if participants took five steps, responded to a condition in their sixth steps, and continued walking until they passed the finishing point.

### Data processing and analysis

Nexus Vicon software was used to analyse individual gait cycles (defined as the heel strike of a foot to the heel strike of the same foot) in each trial in the baseline and adaptability tests. Spatiotemporal parameters (step velocity, stance time, swing time, double support (DS) time, and step length (SL) were computed. Leg length for each participant was used to normalise the previous and target step lengths (the first and second step of the gait cycle). Velocity (m/s) was calculated by dividing SL to the step time (SL/step time). Stance time, swing time and DS time were reported as percentages of a gait cycle. In adaptability tests, the horizontal distance between the toe marker and projected laser lines in the target step were called long/short step length absolute errors (errors), and the vertical distance between the toe marker and the obstacle in the target step was called toe-obstacle clearance height.

### Statistical analysis

All statistical analyses (α = 0.05) were performed in SPSS (Version 25 for Windows, SPSS Science, Chicago, USA). Analyses of variance (ANOVA) repeated measured tests were used to investigate the effects of group (young vs. healthy older vs. older diabetes) and condition (baseline vs. walkthrough vs. short/long/obstacle) on dependent gait parameters (velocity, stance time, swing time, double support time, step length). Multiple comparisons (Bonferroni post hoc tests) were used when the main effect of groups was significant. Nonparametric tests (Kruskal-Wallis H and Mann-Whitney U) were used to compare short/long step length errors and toe obstacle clear heights between groups.

## Results

Forty-three participants (16 persons in Group I, 14 persons in group II, 13 persons in Group III) completed gait adaptability tests. Excluded participants included two in Group II, one in Group III who felt uncomfortable about walking under four conditions, and two in Group III who had a history of fall, which had been excluded from the study because of falls on gait adaptability in previous research [8].

Table 1 presents the characteristics of participants. Older participants in Group II and Group III were significantly older and taller than young participants; however, no significant differences were found between the characteristics of these two groups. Group I differed from Group II and Group III in terms of age (*p* < 0.001 and *p* < 0.001) and height (*p* = 0.034 and *p* = 0.039).

**Table 1:** Descriptive statistics of each group pf participants. Mean ± standard deviation of age, body mass and height in young adults (Group I), healthy older adults (Group II), and older adults with diabetes (Group III) are presented.

	Group I (n = 16)	Group II (n = 14)	Group III (n = 13)
Participants	9 males, 7 females	8 males, 6 females	7 males, 6 females
Age (years)	26.06 ± 4.97	68.36 ± 5.43 ^a^	69.62 ± 4.81 ^a^
Body mass (kg)	75.61 ± 9.05	75.04 ± 9.75	76.67 ± 11.14
Height (cm)	175.00 ± 6.40	167.93 ± 10.84 ^a^	167.23 ± 9.97 ^a^

^a^ Significantly taller and older than Group I (*p* < 0.05).

**Fig 5:**
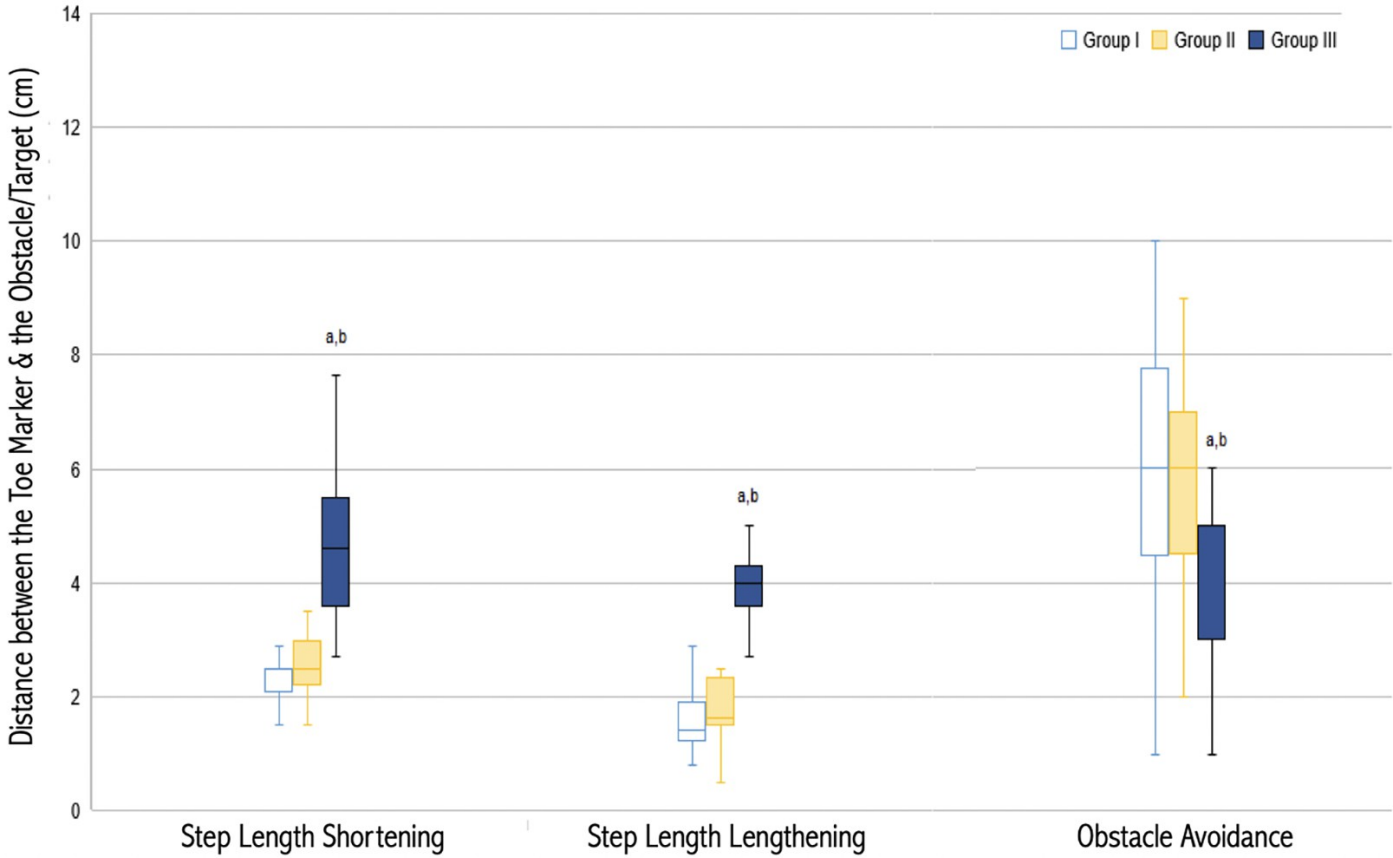
Absolute errors during step shortening, step lengthening, and obstacle clearance adaptation during obstacle crossing in 16 young adults, Group I, 14 healthy older adults, Group II, and 13 older adults with diabetes, Group III. ^a^ Significantly different from the healthy older group (*p* < 0.05). ^b^ Significantly different from the young group (*p* < 0.05).

Fig 5 shows target step length absolute errors and toe-obstacle clearance heights of participants during adaptability tests. Mean absolute errors in response to SSL and LSL targets were different between groups (H (2) = 25.05, *p* < 0.0001, η^2^ = 0.60 and H (2) = 24.13, *p* < 0.0001, η^2^ = 0.59). Absolute errors in the step shortening and step lengthening conditions were different between Group I and Group III (U = 3, p < 0.0001 and U = 5, p < 0.0001) and Group II and Group III (U = 7.5, p < 0.0001 and U = 6, p < 0.0001). Group III, older adults with diabetes, had the largest errors in responses to stepping targets. Between groups, toe-obstacle clearance heights were significantly different (H (2) = 10.03, *p* = 0.007). The mean toe-obstacle clearance height of Group III was significantly shorter than those of both Group I (U = 44, *p* = 0.007) and Group II (U = 33, *p* = 0.004).

Table 2 presents gait parameters in baseline and in adaptability tests in the challenging conditions.

**Table 2:** The comparison of gait and gait adaptability between groups. Mean ± standard deviation (SD) of spatiotemporal parameters in Group I, young adults (n = 16); Group II, healthy older adults (n = 14); and Group III, older adults with diabetes (n = 13) in baseline walking and adaptability test conditions.

Variable	Group	Baseline	Walk-through	Step shortening		Step lengthening		Obstacle crossing	
				Previous step	Target step	Previous step	Target step	Previous step	Target step
Velocity (m/s)	I	1.29 ± 0.13	1.27 ± 0.13	1.25 ± 0.12	1.14 ± 0.10 ^c^^,^^d^	1.28 ± 0.16	1.43 ± 0.15 ^c^^,^^d^	1.23 ± 0.05 ^d^	1.27 ± 0.18
	II	1.25 ± 0.10	1.21 ± 0.11	1.19 ± 0.14 ^d^	1.14 ± 0.18 ^c^^,^^d^	1.24 ± 0.18 ^d^	1.47 ± 0.26 ^c^^,^^d^	1.21 ± 0.26 ^d^	1.28 ± 0.2 ^d^
	III	1.22 ± 0.12	1.08 ± 0.45 ^b^^,^^d^	1.05 ± 0.14 ^a^^,^^b^^,^^c^^,^^d^	0.88 ± 0.17 ^a^^,^^b^^,^^c^^,^^d^	1.11 ± 0.15 ^a^^,^^b^^,^^d^	1.25 ± 0.22 ^a^^,^^b^^,^^d^	1.04 ± 0.28 ^a^^,^^b^^,^^d^	1.01 ± 0.19 ^a^^,^^b^^,^^d^
Stance (%gait)	I	60.71 ± 1.32	60.78 ± 1.04	62.02 ± 1.78	59.01 ± 1.27 ^d^	59.22 ± 1.54	59.01 ± 1.27 ^d^	60.12 ± 1.72	59.90 ± 1.41 ^c^^,^^d^
	II	60.87 ± 1.61	61.32 ± 1.37	62.10 ± 1.69	59.41 ± 1.84 ^d^	59.75 ± 2.31	59.41 ± 2.84 ^d^	60.39 ± 1.73	60.02 ± 1.60 ^c^^,^^d^
	III	61.83 ± 1.81	64.0 ± 1.2 ^a^^,^^b^^,^^d^	63.65 ± 3.57 ^a^^,^^b^^,^^d^	61.15 ± 3.16 ^a^^,^^b^^,^^d^	60.88 ± 2.11 ^c^	61.15 ± 3.16 ^b^^,^^c^	61.42 ± 1.91 ^c^	62.22 ± 2.39 ^a^^,^^b^^,^^c^
Swing (% gait)	I	39.05 ± 1.2	39.43 ± 1.98	-	39.29 ± 1.91	-	43.24 ± 1.90 ^c^^,^^d^	-	41.04 ± 1.34 ^c^^,^^d^
	II	38.10 ± 1.15	38.98 ± 1.80	-	40.20 ± 1.89	-	43.62 ± 2.64 ^c^^,^^d^	-	41.16 ± 1.17 ^c^^,^^d^
	III	38.43 ± 1.79	37.28 ± 2.02 ^b^	-	40.11 ± 3.16 ^c^^,^^d^	-	41.39 ± 2.06 ^b^^,^^c^^,^^d^	-	41.94 ± 1.95 ^c^^,^^d^
Double Support (% gait)	I	10.81 ± 1.1	10.42 ± 1.17	-	10.22 ± 0.88	-	9.51 ± 0.79 ^c^^,^^d^	-	10.03 ± 1.13
	II	10.83 ± 1.09	11.17 ± 1.46	-	10.00 ± 1.43	-	9.87 ± 1.66 ^c^^,^^d^	-	10.04 ± 1.69 ^c^
	III	11.80 ± 1.66	12.03 ± 2.72 ^a^^,^^b^^,^^d^	-	12.34 ± 2.24 ^a^^,^^b^	-	12.26 ± 1.70 ^a^^,^^b^	-	11.61 ± 3.79 ^a^^,^^b^
Step Length (% leg length)	I	78.03 ± 2.99	75.28 ± 4.57 ^d^	70.89 ± 6.53 ^c^^,^^d^	59.78 ± 6.9 ^c^^,^^d^	78.61 ± 5.77 ^c^	98.03 ± 5.77 ^c^^,^^d^	72.73 ± 4.16 ^d^	78.22 ± 5.62 ^c^
	II	77.37 ± 6.33	73.49 ± 6.67 ^d^	66.39 ± 4.48 ^c^^,^^d^	60.97 ± 4.72 ^c^^,^^d^	77.92 ± 7.01 ^c^	97.2 ± 6.11 ^c^^,^^d^	69.66 ± 11.62 ^d^	80.87 ± 8.54 ^c^^,^^d^
	III	74.21 ± 6.99	70.26 ± 5.28 ^b^^,^^d^	67.39 ± 4.09 ^b^^,^^c^^,^^d^	66.88 ± 7.29 ^a^^,^^b^^,^ ^c^^,^^d^	79.09 ± 7.19 ^c^	95.77 ± 7.2 ^c^^,^^d^	69.19 ± 12.27 ^d^	78.21 ± 9.64 ^c^^,^^d^

a Significantly different from the healthy older group (p < 0.05).

b Significantly different from the young group (p < 0.05).

c Significantly different from the walk-though condition (p < 0.05).

d Significantly different from the baseline condition (p < 0.05).

### Step shortening condition

Conditions were significantly different in: target step velocity (*F*_1.65, 65.98_ = 50.66, *p* < 0.001); previous step velocity (*F*_1.65, 66.33_ = 19.17, *p* < 0.001); leading leg stance time (*F*_2,80_ = 14.51, *p* < 0.001); swing time (*F*_2,80_ = 6.422, *p* = 0.003); DS time (*F*_2,80_ = 6.97, *p* = 0.002); target step length (*F*_1.69, 67.85_ = 109.68, *p* < 0.001); and previous step length (*F*_2,80_ = 63.76, *p* < 0.001).

Groups were significantly different in: target step velocity (*F*_2, 40_ = 10.64, *p* < 0.001); previous step velocity (*F*_2,40_ = 7.11, *p* = 0.002); trailing leg stance time (*F*_2,40_ = 6.85, *p* = 0.003); leading leg stance time (*F*_2,40_ = 6.95, *p* = 0.003); DS time (*F*_2,40_ = 9.29, *p* < 0.001,); previous step length (*F*_2,40_ = 3.00, *p* = 0.047); and target step length (*F*_2,40_ = 4.51, *p* = 0.017).

### Step lengthening condition

Conditions were significantly different in: target step velocity (*F*_1.56, 62.47_ = 44.07, *p* < 0.0001); trailing leg stance time (*F*_2,80_ = 21.24, *p* < 0.0001); leading leg stance time (*F*_1.35,54.11_ = 4.39, *p* = 0.027); swing time (*F*_1.59,63.78_ = 66.11, *p* < 0.0001), DS (*F*_2,80_ = 22.30, *p* < 0.0001); previous step length (*F*_1.86,74.73_ = 16.53, *p* < 0.0001); and target step length (*F*_1.44,57.67_ = 45.26, *p* < 0.0001).

Groups were significantly different in: previous step velocity (*F*_2, 40_ = 5.84, *p* = 0.006); target step velocity (*F*_2, 40_ = 5.59, *p* = 0.007); trailing leg stance time (*F*_2,40_ = 59.08, *p* = 0.004); swing time (*F*_2,40_ = 3.88, *p* = 0.029); and DS time (*F*_2,40_ = 8.26, *p* = 0.001).

### Obstacle crossing condition

Conditions were significantly different in: previous step velocity (*F*_1.73, 69.36_ = 17.10, *p* < 0.0001); target step velocity (*F*_1.60, 64.12_ = 6.66, *p* = 0.004); trailing leg stance (*F*_2, 80_ = 9.40, *p* < 0.0001); leading leg stance (*F*_1.68, 67.45 =_ 12.22, *p* < 0.0001); swing time (*F*_2, 80_ = 39.64, *p* < 0.0001); DS time (*F*_2, 80_ = 15.014, *p* < 0.0001); target step length (*F*_1.89, 75.68_ = 42.00, *p* < 0.0001); and previous step length (*F*_1.20, 48.15_ = 8.17, *p* = 0.004).

Groups were significantly different in previous step velocity (*F*_2, 40_ = 6.27, *p* = 0.004); target step velocity (*F*_2, 40_ = 7.63, *p =* 0.002); trailing leg stance (*F*_2, 40_ = 8.77, *p* = 0.001); and DS time (*F*_2, 40_ = 7.12, *p* = 0.002). Previous step velocity in older adults with T2D was different from other groups (*p* = 0.004 and *p* = 0.009).

## Discussion

This study included three groups of participants to differentiate the effects of ageing, T2D, and the combination of T2D and ageing using novel combined paradigms of virtual stepping targets and an obstacle. For the first time, participants who reported a history of falls and had neuropathy were excluded from data analyses. The results of tests showed that older adults with T2D had normal gait in baseline when no challenge was included; however, they had impaired gait adaptability, increased step length errors, and reduced toe-obstacle clearance heights in the challenging conditions.

In line with previous research [27–31], ageing and T2D did not affect gait characteristics during walking at a preferred speed when no challenge was involved. In a study reporting gait impairments [28], experimental groups were different in characteristics such as history of falls, cognition, and the distribution of gender or walking speed [31]. Even though groups were different in these characteristics, they might not show any significant differences because locomotion was unchallenged; therefore, afferent information may not be required to update efferent copies of the locomotion in the central nerve system [32, 33]. When a person walks on a smooth surface at a preferred speed without any challenge, the central nervous system adapts gait in a way that muscles consume the minimum amount of energy. Since using receiving afferents can take too much time in keeping track of the current state, the system uses efferent copies of locomotion at the level of the spinal cord as a baseline for information about the task [34].

In walk-through condition, when the task was to walk through without adapting sagittal foot displacement trajectories, some gait parameters significantly differed from those in baseline. In line with the finding of previous research [8], participants reduced their step velocities and walked with shorter steps, longer DS, and stance times, which might be interpreted as being ready to suddenly respond to a condition. Like other conditions in adaptability tests, the walk-through condition might increase the load of attention and as such affect gait compared with the baseline condition. This made participants be ready to suddenly change their ongoing gait and find an alternative foot landing position to meet the presented tasks.

Having conservative gait patterns in the walk-through condition of adaptability tests, the older adults with T2D adapted gait parameters in the previous step in response to the step shortening condition. They reduced velocity, increased DS time, reduced the previous step length, and then shortened their target step. However, the other groups only reduced the velocity and length of the previous step. The earlier changes in the previous step in older diabetics were more pronounced, revealing that they were more affected in the adaptability tests. Our results of perturbing step lengths (i.e., presenting SSL targets) did not confirm differences in errors between healthy older and young adults, which disagrees with previous research reports [8] where some of the participants had experienced one or two falls a year before participation. In this study, fall history might have increased the error of step shortening, but was excluded as a criterion in our research project. Our older participants had no history of falls and were physically fit; they walked as fast as young adults with a similar SL during overground walking.

Although older adults with T2D increased the step length of both previous and target steps in the step lengthening condition, they made the largest errors in the step lengthening condition compared with other groups. The healthy older and young groups increased the target step lengths, whereas the older diabetic group increased the swing time of the target step and increased the step lengths of the previous and target steps. To process appropriate responses, older adults with T2D reduced the velocity of the previous step and increased the stance time of the leading foot. The older with and without T2D reduced the previous step velocity when they detected the task. Healthy older adults increased the target step velocity to respond to the task; however, older adults with T2D were unable to increase the velocity of the target step as much as the healthy older adults. Thus, they had to increase the stance time of the trailing foot in the previous step to match the toe marker with a laser beam in the target step without being able to lengthen steps accurately.

Older adults with T2D showed reduced toe-obstacle clearance height when they responded to the obstacle crossing condition. They reduced step velocities and increased the DS and stance times in response to the obstacle crossing condition. However, consistent with previous research [33], these modulations of gait parameters reduced the toe-obstacle clearance height. Using a real obstacle with other conditions made the prediction of triggering the obstacle too difficult. Participants had to cross the obstacle and could not use any other strategy such as adding a short step prior obstacle avoidance as reported as a compensatory strategy in previous research [8]. Therefore, older adults with T2D showed a reduced toe-obstacle clearance height. Diabetes might reduce the reaction time and the accuracy of responses to stimuli as efferent patterns in the central nervous system might be unable to be updated because the afferent feedback was inadequate [34]. Reduced step velocities in both previous and target steps and increased DS time in older adults with T2D increased the time of responding to goal tasks compared with other groups, as reported in previous research [35]. However, they were unable to have a comparable toe-obstacle clearance height with other groups.

In interpreting the results of the present study, it is important to postulate why older individuals with diabetes might have a quantifiably more impaired gait adaptability compared to both older and healthy counterparts, despite excluding individuals with diabetic neuropathy from the present study. Previous research has identified the diabetic population to be at increased risk of falls due to proposed multifactorial contributions including impaired vision and visual-motor coordination, polypharmacy affecting gross motor function, and a comparatively sedentary lifestyle [2]. It is interesting, however, in the present study, that biomechanical differences in gait adaptability were not noted at baseline but only during obstacle avoidance challenges. Thus, one could postulate that the pathophysiology of T2D and poor glycaemic control affects an individual’s central nervous system and motor control pathways in ways that manifest functionally as an impaired ability to adapt movement in real-time. In previous studies [8, 15], participants with diabetic neuropathy and a history of falls were not excluded and remained us with a doubt whether the findings would be different if these participants were excluded. By excluding individuals with established diabetic neuropathy, the present study demonstrates that biomechanical changes in gross motor function can be identified in diabetic individuals before disease progression to diabetic neuropathy.

This study has several limitations. The choice of parameters for investigation was limited to the sagittal plane. Foot placement adjustments were not investigated in the mediolateral direction since the overground gait adaptability tests could not present targets for investigating the mediolateral foot placement adjustments. Finally, this study’s assessments were designed to improve upon previous assessments of the clinical population’s ability to adapt gait by introducing targets and obstacles in real-time rather than analysing individual’s obstacle avoidance strategies for obstacles that were visible from a starting point and could be planned for accordingly [15, 16]. Despite this improvement in experimental design, the study was indeed conducted in the controlled environment of a gait laboratory, and thus participants’ knowledge of gait being studied with impending obstacles may complicate the generalizability of gait adaptability differences in real-world walking.

Further, future studies may investigate how the extent of disease progression in diabetes is correlated to the biomechanical gait changes identified in this study, using measures such as haemoglobin A1C, level of glycaemic control, or medications used.

In conclusion, this study has demonstrated that the interaction of ageing and T2D leads to impaired gait adaptability and more conservative gait patterns in older adults with T2D. The presented test design can be used as a tool to identify people at the risk of tripping/falling. Future study is required to use the test setup for identifying whose gait adaptability is impaired. Impaired gait adaptability may place older adults with T2D at an increased risk of falls while reacting to unexpected challenges during walking. Therefore, some training programs [36–38] can be used to assist this population with reducing the occurrence of touching obstacles during walking.
